# Croup review: comparative analysis of acute and recurrent croup

**DOI:** 10.36416/1806-3756/e20240353

**Published:** 2024-11-16

**Authors:** Sofia Prates da Cunha de Azevedo, Laura Gomes Boabaid de Barros, Júlia Giffoni Krey, Leonardo Araújo Pinto, Sérgio Luís Amantéa

**Affiliations:** 1. Grupo de Pesquisa em Epidemiologia e Genética das Doenças Respiratórias da Infância, Escola de Medicina, Pontifícia Universidade Católica do Rio Grande do Sul - PUCRS - Porto Alegre (RS) Brasil.; 2. Programa de Pós-Graduação em Medicina/Pediatria, Escola de Medicina, Pontifícia Universidade Católica do Rio Grande do Sul - PUCRS - Porto Alegre (RS) Brasil.; 3. Núcleo de Pediatria, Escola de Medicina, Pontifícia Universidade Católica do Rio Grande do Sul - PUCRS - Porto Alegre (RS) Brasil.; 4. Departamento de Pediatria, Faculdade de Medicina, Universidade Federal de Ciências da Saúde de Porto Alegre - UFCSPA - Porto Alegre (RS) Brasil.

Croup is a common respiratory illness of the larynx, trachea, and bronchi which is manifested by stridor and a barking cough. Laryngotracheitis and laryngotracheobronchitis have been included in the croup spectrum. It involves the narrowing of the laryngeal lumen and subglottic region, leading to airway inflammation and edema.^1^ Croup is typically self-limited, occurring predominantly during the fall and winter. It is more common in boys than in girls (1.5:1 ratio). Croup affects approximately 3% of children between six months and six years of age.^1^ However, it can occur in children up to six years of age, or even before six months.^2^ ^-^ ^4^

Most patients present with mild conditions (less than 5% requiring hospitalization), and, of these, less than 3% may require tracheal intubation. Parainfluenza virus (types 1 to 3) accounts for 75% of all cases, and human parainfluenza virus 1 is the most common type. Other viral etiologies include influenza A and B, adenovirus, respiratory syncytial virus, rhinovirus, enterovirus and SARS-CoV-2. Spasmodic croup is also caused by the same viruses, but lacks signs of infection. Bacterial causes are rare. *Staphylococcus aureus, Streptococcus pneumoniae, Haemophilus influenzae,* and *Moraxella catarrhalis* may be involved.^2^ ^,^ ^5^ ^,^ ^6^

Viral croup often presents similarly to an upper respiratory infection, with 12-72 h of low-grade fever and coryza. Narrowing of the larynx leads to stridor, increased respiratory rate, respiratory retractions, and a barking cough. Symptoms worsen at night and peak between 24 and 48 h. In most cases it improves spontaneously within 48 h to a week. Croup is a clinical diagnosis, and there is no need for additional tests to confirm it in most cases.^2^ ^,^ ^5^

In patients with recurrent croup (more than two episodes per year), bronchoscopic abnormalities may be associated with risk factors: previous intubation, prematurity, and young age. Gastroesophageal reflux disease, asthma, and atopy are also more prevalent in recurrent croup, but do not usually show abnormalities at bronchoscopy.^2^ Chart 1 shows useful clinical manifestations for the differential diagnosis of obstructive diseases of the upper airway.^2^ ^,^ ^7^

**Chart 1:** Clinical manifestations, differential diagnosis, and management recommendations of croup in children.

MANIFESTATIONS AND DIFFERENTIAL DIAGNOSIS		
Etiology	Age range	CLINICAL MANIFESTATIONS
Croup	6 months to 3 years	Acute onset of barking cough, stridor, and hoarseness
Bacterial tracheitis	< 6 years	High fever, barking cough, respiratory distress, and rapid deterioration
Congenital or acquired airway lesions	< 6 months to 4.5 years	Recurrent episodes of barking cough and stridor
Foreign body aspiration	< 3 years	Acute onset of choking and/or drooling
Hemangioma	< 6 months	Stridor worse with crying
Laryngomalacia	< 18-24 months	Stridor that worsens with crying and feeding. Symptoms related to position.
Neoplasm	No age predilection	Progressive airway symptoms
Retropharyngeal, parapharyngeal, peritonsillar abscesses	6 months to 35 years	High fever, neck pain, sore throat, and dysphagia followed by torticollis, drooling, and stridor
MANAGEMENT		
ACUTE MANAGEMENT	• Corticosteroids administered orally, inhaled, or intramuscularly • Nebulized epinephrine may be recommended for moderate to severe airway obstruction	
RECURRENT CROUP MANAGEMENT	• Endoscopic airway evaluation may help identification of underlying airway disorder • In the acute management of recurrent croup, patients may receive the standard treatment above >• Inhaled steroids may be used to reduce recurrence	

Management of croup is based on the severity of illness. Clinical signs of level of consciousness, as well as presence of cyanosis, stridor, air entry, and retractions, have been used to structure severity scores. However, they are not mandatory in the clinical context. They can be useful when the team has less expertise and experience. More recently, the incorporation of the number of doses of racemic epinephrine in the emergency department (ED), previous administration of dexamethasone, and history of intubation have been used to improve the predictive accuracy of the need for hospitalization.^1^ ^,^ ^2^ ^,^ ^7^

The management of acute viral croup includes corticosteroids that may be administered orally, inhaled, or intramuscularly, considering different criteria as the severity of the condition. However, further studies are necessary to elucidate the potential dose-dependent effects of the medication and to evaluate the benefits of administering multiple doses to children assisted in the ED. Additionally, nebulized epinephrine can be prescribed as it is recommended for cases of moderate to severe upper airway obstruction, characterized specially by increased difficulty of breathing. The effects of mild viral croup are usually transient, and epinephrine does not provide sustained benefits in cases with mild symptoms.^2^ ^,^ ^5^ ^,^ ^7^

Historically, recurrent croup had been considered an anatomical issue related to airway abnormalities, prompting evaluations with laryngoscopy or bronchoscopy procedures under anesthesia. However, recently experts described that it resembles airway reactivity similar to asthma.^(8 )^ Previously, inhaled corticosteroids (ICS) were used for acute croup episodes because of suggested benefits as a preventative therapy. In a large cohort study,^5^ it was hypothesized that prophylactic ICS could potentially decrease both the frequency and severity of recurrent croup episodes in patients with no fixed airway lesions. Another study^6^ retrospectively reviewed charts of children referred to outpatient clinics for recurrent croup between June of 2019 and January of 2021. In that study, recurrent croup was defined as three or more episodes occurring within a lifetime.^6^

Among the patients who underwent imaging or diagnostic laryngoscopy/bronchoscopy, there were few airway abnormalities, and none required surgical intervention. Most patients treated with medical therapy used fluticasone propionate inhalers twice daily upon the onset of an upper respiratory infection. Nearly 90% of parents reported improvement in symptoms. There were no significant differences in past medical history or comorbidities between patients who improved on ICS and those who did not, and no reported adverse drug reactions. ICS treatment seemed to be particularly effective in patients with more than five episodes of croup. In addition, some patients with gastroesophageal reflux disease or eosinophilic esophagitis also reported improvements with ICS therapy.^6^ The initiation of ICS at the first sign of a viral upper respiratory infection in order to reduce episodes of recurrent croup is a relatively a novel preventative treatment. It is necessary to conduct randomized control trials to validate its effectiveness in the future.

## CONCLUSION

Although croup is a condition that is often resolved on its own, it can be a difficult illness to deal with due to the need of regular doctor visits and usage of health care resources. Further research is needed, and it is likely that early intervention with oral or inhaled corticosteroids will still be the primary approach to croup management, given its importance in lowering mortality and morbidity.

## References

[1] Quraishi H, Lee DJ (2022). Recurrent Croup. Pediatr Clin North Am.

[2] Smith DK, McDermott AJ, Sullivan JF (2018). Croup: Diagnosis and Management. Am Fam Physician.

[3] Sowa LE, Stillwell PC, Houin PR, Nguyen N, Prager JD, Wine T (2023). Prophylactic inhaled corticosteroids for the management of recurrent croup. Int J Pediatr Otorhinolaryngol.

[4] Garzon Mora N, Jaramillo A P, Briones Andriuoli R, Torres S, Revilla JC, Moncada D (2023). An Overview of the Effectiveness of Corticoids in Croup: A Systematic Literature Review. Cureus.

[5] Petrocheilou A, Tanou K, Kalampouka E, Malakasioti G, Giannios C, Kaditis AG (2014). Viral croup diagnosis and a treatment algorithm. Pediatr Pulmonol.

[6] Tyler A, Bakel LA, Tucker J, Moss A, Kille B, Rifken K (2024). Health Services Use for SARS-CoV-2-Infected Children With Croup or Bronchiolitis. Hosp Pediatr.

[7] Ortiz-Alvarez O (2017). Acute management of croup in the emergency department. Paediatr Child Health.

